# Nitric oxide in the cardiovascular system: a simple molecule with complex actions

**Published:** 2009-10

**Authors:** Hans Strijdom, Nontuthuko Chamane, Amanda Lochner

**Affiliations:** Division of Medical Physiology, Department of Biomedical Sciences, Faculty of Health Sciences, Stellenbosch University, South Africa; Division of Medical Physiology, Department of Biomedical Sciences, Faculty of Health Sciences, Stellenbosch University, South Africa; Division of Medical Physiology, Department of Biomedical Sciences, Faculty of Health Sciences, Stellenbosch University, South Africa

## Abstract

**Summary:**

Since it was identified as the elusive endothelium-derived relaxing factor (EDRF) in the 1980s, nitric oxide (NO) has rapidly gained status as one of the most important signalling molecules in the cardiovascular system. Now, 20 years later, NO is regarded by most to be a ubiquitous mediator of cardio-protection. However, due to various complex underlying cellular mechanisms, the actions of NO often seem to be contradictory. This article sheds light on some of the mechanisms that may influence the variable actions of NO in the heart. Its role in conditions of oxygen deprivation (ischaemia and hypoxia) in particular is relevant to basic scientists and clinicians alike, since the prevalence of ischaemic heart disease is on the rise (in both the developed and the developing worlds) and novel therapeutic options are in constant demand. NO is a promising candidate molecule that could find therapeutic application. For this to be achieved, a sound understanding of this simple molecule and its complex actions is required.

## The discovery of nitric oxide: a simple molecule with a wide range of biological effects

The groundbreaking discovery in 1987, that a previously unidentified molecule, rather loosely termed ‘endothelium-derived relaxing factor’ (EDRF), was in fact nitric oxide (NO), caused a paradigm shift in scientists’ and clinicians’ understanding of cardiovascular physiology and pathophysiology.^1^ The notion that a molecule, until then regarded as a toxic air pollutant, could be endogenously produced and could play a role as a major cardiovascular signalling molecule in mammals was indeed surprising, if not sensational at the time and resulted in the 1998 Nobel Prize for Medicine being awarded to the researchers involved.

This discovery also culminated in an understanding of the previously unknown mechanism of action of nitroglycerine (now known to be a NO-releasing compound), a popular antiangina pectoris drug prescribed by clinicians since the early 20th century! Today NO is regarded as one of the most important mediators of biological processes in the heart and blood vessels. However, the biological effects of NO are variable, and this fact is becoming increasingly evident as our knowledge of this wonder molecule expands.

NO is a simple diatomic gas and free radical that is endogenously synthesised by a family of enzymes called NO synthases (NOS).^2^,^3^ NOS are expressed in a variety of tissues throughout the body, and are particularly prominent in the nervous and cardiovascular systems.^4^ Since it is a free radical, NO can react with a large number of molecules in the body,^5^,^6^,^7^ and the fact that it is a gas allows for easy passage between cells and tissues.^8^ These biochemical properties not only enable NO to be an ideal signalling molecule, but also result in a wide range of (often contradictory) biological effects (See Table 1 for a summary of the biological effects of NO in the cardiovascular system).

**Table 1:** Biological Effects Of NO In The Cardiovascular System

*Target cells/tissue/organ*	*Effect*
Vasculature	
Smooth muscle cells	Relaxation → vasodilatation
Platelets	Anti-platelet aggregation
Inflammatory cells	Anti-inflammatory actions
Reactive oxygen species	Anti-oxidant effects
Endothelial cells	Angiogenesis
Heart	
Myocardium	Foetal and postnatal growth and development
	↑↓ Contractile function
	Anti-hypertrophy
	Cardioprotective against ischaemic injury
	Cell generation and proliferation
	Anti-apoptotic; pro-survival
	↑↓ contraction
	Anti-hypertrophy
Cardiomyocytes	Harmful when present in excessive amounts: pro-apoptotic, pro-necrotic

**Fig. 1.**
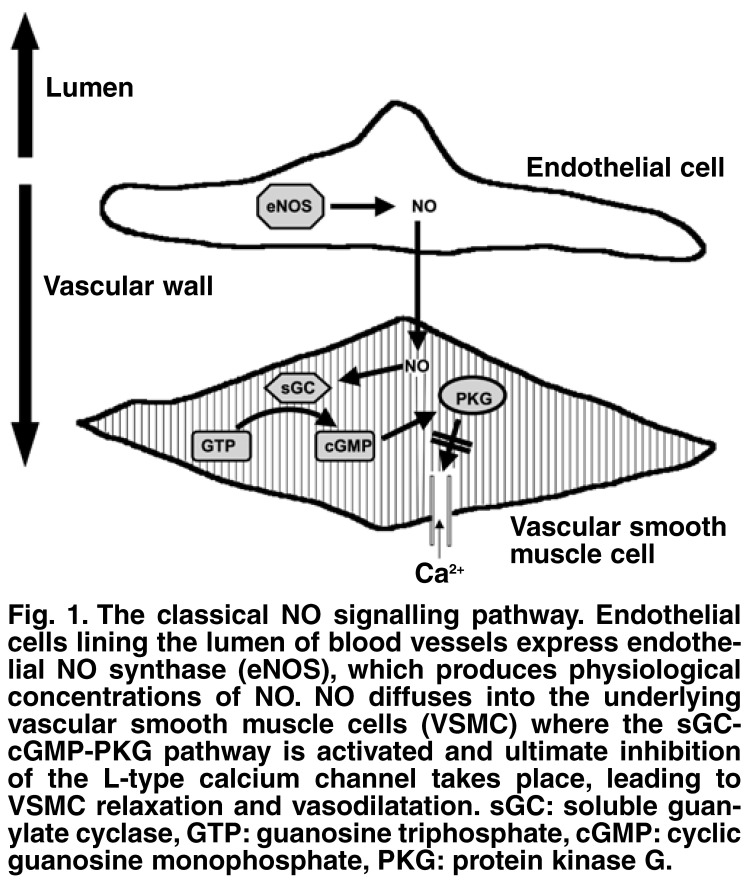
The classical NO signalling pathway. Endothelial cells lining the lumen of blood vessels express endothelial NO synthase (eNOS ), which produces physiological concentrations of NO . NO diffuses into the underlying vascular smooth muscle cells (VSMC) where the sGCcGM P-PKG pathway is activated and ultimate inhibition of the L-type calcium channel takes place, leading to VSMC relaxation and vasodilatation. sGC: soluble guanylate cyclase, GT P: guanosine triphosphate, cGM P: cyclic guanosine monophosphate, PKG: protein kinase G.

The role of NO in the maintenance of vascular homeostasis is well defined; a role that relates to the original discovery that endothelium-derived NO diffuses into underlying vascular smooth muscle cells where the classical NO-cGMP-protein kinase G (PKG) signalling pathway causes vascular relaxation (Fig. 1). Generally speaking, NO promotes a vasodilatory, anti-thrombotic and anti-inflammatory state in the vasculature; however, when the bioavailability of NO is compromised, these beneficial actions are lost and endothelial dysfunction ensues.^9^

## NO in the heart

In the heart, the role of NO seems less well defined^10^ and is, to the frustration of many researchers, often characterised by contradictory experimental observations in the physiological as well as pathophysiological setting (such as low oxygen supply: hypoxia/ischaemia).^3^,^11^ Although many of these contradictory findings can be explained by technical factors such as differences in experimental models and protocols, there are several physiological factors that should be considered in understanding the behaviour of NO. These are discussed below.

## Enzymatic sources of NO

The primary cellular source of NO, NOS are a family of three known isoforms, namely, neuronal NOS (nNOS, NOS-1), inducible NOS (iNOS, NOS-2) and endothelial NOS (eNOS, NOS-3).^4^ All three isoforms are expressed in the heart.^4^ Both eNOS and nNOS are constitutively expressed, low-output enzymes and eNOS, in particular, is associated with the maintenance of basal, physiological cardiac function.^12^ The expression of the third isoform, iNOS, is dependent on induction by cytokines such as TNF-α or other pathophysiological stimuli.^4^ iNOS is a high-output enzyme and generates up to 1 000-fold more NO than eNOS.^2^ iNOS-derived NO can lead to harmful effects, not due to the direct actions of NO *per se*, but rather to the abundance of NO that becomes available in such a setting to react with superoxide radical (O_2_-), leading to the formation of the highly reactive (and harmful) radical, peroxynitrite (ONOO-), and further downstream, derivatives such as nitryl and hydroxyl.^13^

Contractile function is also influenced by the amount of NO; at low (submicromolar) doses, there seems to be a small positive inotropic effect, whereas higher (micromolar or above) doses have negative inotropic effects.^14^,^15^ Therefore, the biological effects of NO in the heart can vary greatly depending on which NOS isoform is activated and the amount of NO released. This is further confounded by the differential expression of the NOS isoforms in various cardiac cell types: eNOS expression is highest in the endocardial and cardiac microvascular endothelial cells,^16^ whereas iNOS expression has been shown to be relatively higher in cardiomyocytes.^3^

## Subcellular localisation of NOS

**Fig. 2.**
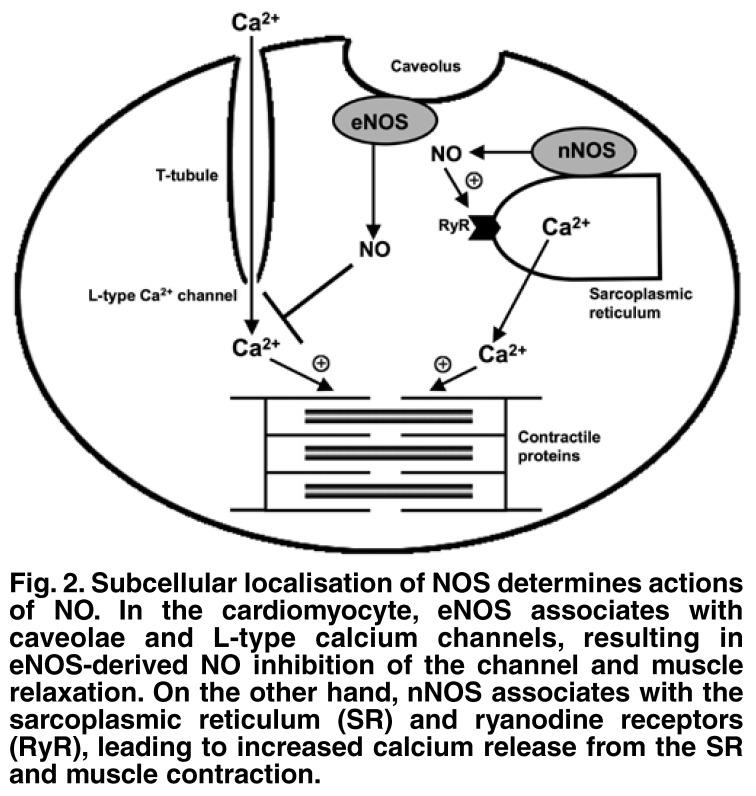
Subcellular localisation of NOS determines actions of NO . In the cardiomyocyte, eNOS associates with caveolae and L-type calcium channels, resulting in eNOS-derived NO inhibition of the channel and muscle relaxation. On the other hand, nNOS associates with the sarcoplasmic reticulum (SR ) and ryanodine receptors (RyR), leading to increased calcium release from the SR and muscle contraction.

Beta-adrenergic stimulation of hearts of eNOS-/- knockout mice had positive inotropic effects, whereas in hearts of nNOS-/- knockout mice, negative inotropic effects were observed.^17^ The underlying mechanism of these apparent paradoxical effects of NO is thought to relate to the subcellular location of these two isoforms of NOS.^8^ Therefore, the spatial confinement of specific NOS isoforms to distinct locations in cardiac cells has important implications for the effects of NO on contractile function (Fig. 2).

In cardiomyocytes, eNOS associates intimately with caveolae (flask-shaped invaginated regions of cell membranes). Caveolae are specialised hubs of high signalling activity, associated with, among others, β-adrenergic receptors and L-type Ca^2+^ channels. The co-localisation of eNOS, β-receptors and Ca^2+^ channels allows eNOS-generated NO to be within diffusion distance of its molecular targets.^17^ The net result of this interaction is a negative inotropic effect, since NO prevents the opening of the Ca^2+^ channels and therefore inhibition of β-adrenergic-induced inotropy. Conversely, nNOS localises to the sarcoplasmic reticulum (SR) and ryanodine receptors (RyR); therefore nNOS-generated NO can readily activate RyR to release Ca^2+^ from the SR and cause a positive inotropic effect, thus mediating an effect on myocardial contractile function, which is directly opposite to that observed with eNOS.^17^

The spatial confinement hypothesis of eNOS and nNOS is constantly undergoing modifications as our knowledge increases. In a recent review article,^10^ it was speculated that nNOS co-immunoprecipitates with xanthine oxidase (XO), an O_2_-generating enzyme, leading to the possibility that nNOS-derived NO combines with O_2_- to form peroxynitrite (ONOO-), and that the latter has direct modulating effects on RyR, phospholamban and even the L-type calcium channels. Conversely, it was suggested that eNOS co-immunoprecipitates with superoxide dismutase (SOD), an O_2_- scavenger, which possibly leads to a negative regulation of the ONOO-generating reaction.

## NOS substrate and co-factor availability

NOS are tightly controlled enzymes, especially at the level of NO synthesis, since NO is a gas that can readily diffuse and therefore cannot be stored in vesicles.^8^ For this reason, NOS are complex enzymes with many regulating co-factors and associated proteins. When there is limited supply or absence of substrate (l-arginine) or co-factors, such as tetrahydrobiopterin, NO production is uncoupled from NADPH oxidation,^9^,^18^ and the enzyme generates O_2_- at the expense of NO. This can explain the paradoxical findings in cells subjected to oxidative stress (an important cause of eNOS uncoupling) when increased eNOS expression is observed without evidence of concomitant increase in NO production.^9^ In the vascular system, eNOS uncoupling is regarded as an important mechanism for the development of endothelial dysfunction.^9^

## Redox status/antioxidant capacity

The generation of reactive oxygen species (ROS) such as O_2_- is regarded as a normal physiological process, provided sufficient antioxidant mechanisms are available. When the ROS scavenging properties of a cell are compromised, such as reduced activity or expression of superoxide dismutase (SOD), unscavenged O_2_- will react with NO (for which it has a high affinity) to form ONOO-, a highly reactive cytotoxic radical, which opposes the effects usually associated with NO.^13^ A similar scenario develops when high amounts of NO are produced by iNOS, as described earlier.

## Non-enzymatic sources of NO

When evaluating the effects of NO on the heart, it should be borne in mind that NOS-independent cellular reactions are able to generate NO. When cellular acidosis develops, such as during ischaemia, nitrites (breakdown products of NO metabolism) can be readily reduced by XO to again form NO, thereby serving as a significant source of biologically active NO.^19^,^20^,^21^ It is therefore quite possible that NO is generated and effects are observed despite down-regulated or inactivated NOS.

**Fig. 3.**
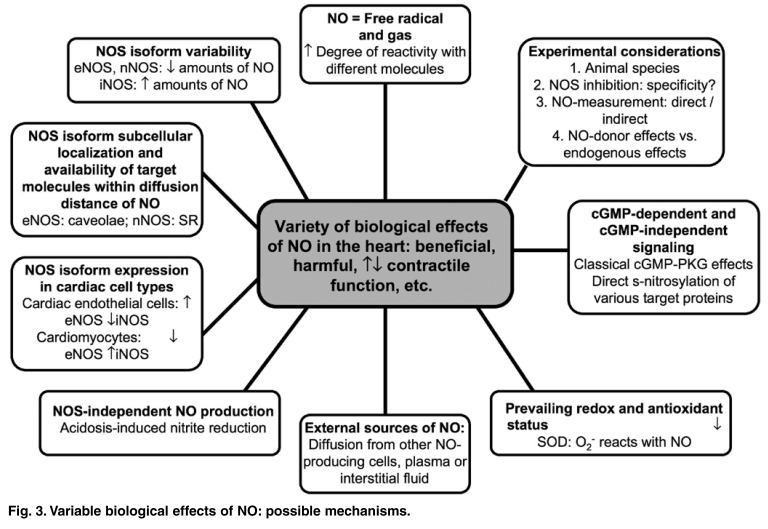
Variable biological effects of NO: possible mechanisms.

From the above, it is clear that there are many underlying mechanisms and physiological factors (see Fig. 3 for summary) that influence the biological actions of NO. The resulting complexity and variability of the effects and the difficulties they often create in the interpretation of findings make further research into the cellular mechanisms of this vital cardiovascular signalling molecule imperative.

## NO and cardiac hypoxia/ischaemia

The role of NO in the myocardium during conditions of low oxygen supply has become a fast-growing field of interest in basic cardiovascular research.^22^ From the literature, it appears that the majority of evidence points to a protective/beneficial role for NO against the injurious effects of myocardial ischaemia, which should be of great interest to clinicians constantly in search of new cardioprotective therapies. In fact, Jones and Bolli, pioneers of research into the role of NO in cardioprotection elicited by ischaemic preconditioning, made the following bold statement in a 2006 review: ‘... whether NO is beneficial or detrimental to the ischemic myocardium is no longer an issue and the basic premise (of this mini-review) is undemanding: NO protects the heart against ischemia-reperfusion injury.’^23^

It is a fact, however, that many authors did observe deleterious effects associated with ischaemia/hypoxia-induced NO production. Despite opinions such as the one expressed by Jones and Bolli, one cannot ignore evidence to the contrary, even if the findings demonstrating harmful effects are in the minority. The possible causes of the apparent paradoxical nature of NO’s biological actions (discussed earlier and summarised in Fig. 3) are as relevant in the pathophysiological setting (i.e. myocardial hypoxia/ischaemia) as they are in the physiological situation. Therefore, contradictory or unexpected findings should always be interpreted with these mechanisms in mind.

## Hypoxia and ischaemia induce increased NO production

One of the first steps in the assessment of the role of any molecule in a pathophysiological setting is to establish whether the concentration of that particular molecule increases or decreases. In this regard, NO is a particularly difficult molecule to study due to its free-radical and gaseous nature and its relatively short half-life. Very few sensitive, specific, reliable and affordable detection techniques exist that are able to directly and quantitatively measure NO levels.^24^ Therefore, many studies investigating NO in cardiac hypoxia/ischaemia have to rely on indirect techniques such as determination of NOS activity (measurement of citrulline level, which is co-produced with NO), nitrate + nitrite levels (breakdown products of NO metabolism), and cGMP levels (second messenger in the NO-sGC-pathway).

**Fig. 4.**
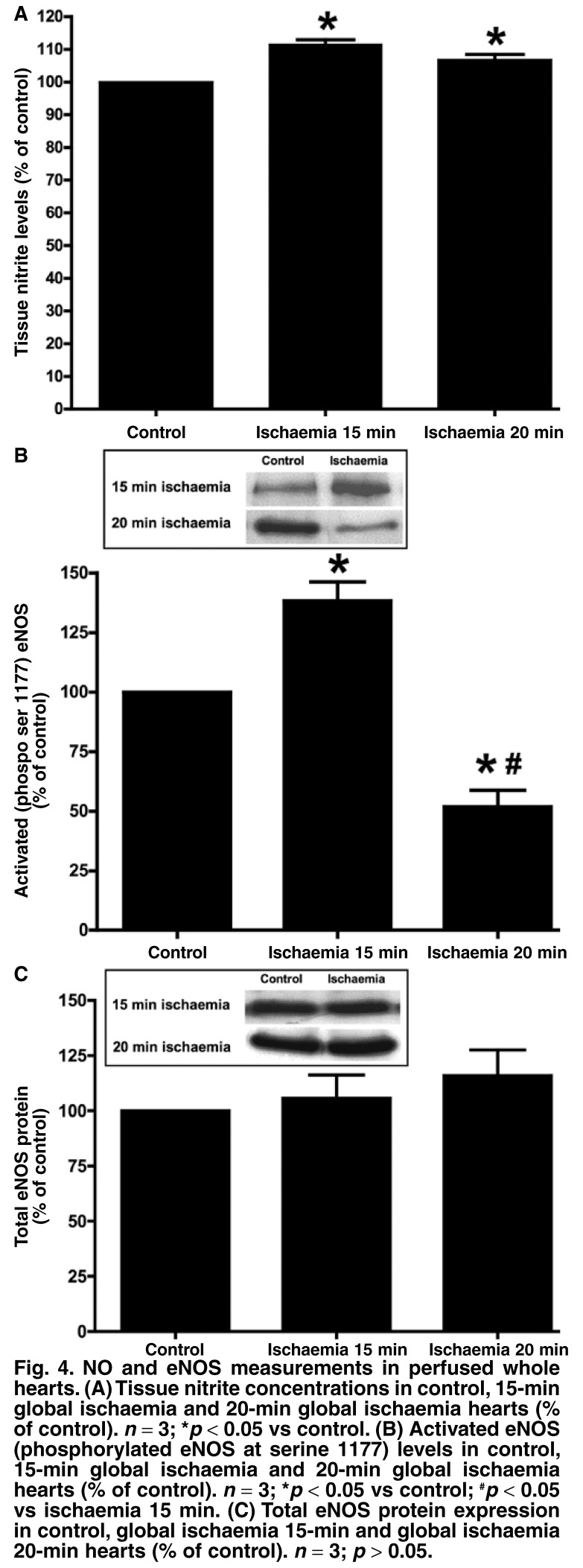
NO and eNOS measurements in perfused whole hearts. (A) Tissue nitrite concentrations in control, 15-min global ischaemia and 20-min global ischaemia hearts (% of control). *n* = 3; *p < 0.05 vs control. (B) Activated eNOS (phosphorylated eNOS at serine 1177) levels in control, 15-min global ischaemia and 20-min global ischaemia hearts (% of control). *n* = 3; *p < 0.05 vs control; ^#^*p* < 0.05 vs ischaemia 15 min. (C) Total eNOS protein expression in control, global ischaemia 15-min and global ischaemia 20-min hearts (% of control). *n* = 3; *p* > 0.05.

Despite the technical challenges, evidence overwhelmingly points to an increase in NO levels, at least during the early stages of hypoxia and ischaemia (in the absence of reperfusion).^3^,^25^-^27^ Our own work also showed elevation of NO production in cardiac cell models subjected to hypoxia, namely, isolated adult rat ventricular cardiomyocytes^24^,^28^ and cardiac microvascular endothelial cells,^28^,^29^ using a specific fluorescent probe that detects intracellular NO (diaminofluorescein, DAF-2/DA), as well as adult whole rat heart models by measuring tissue nitrite concentrations (Fig. 4A).

**Fig. 5.**
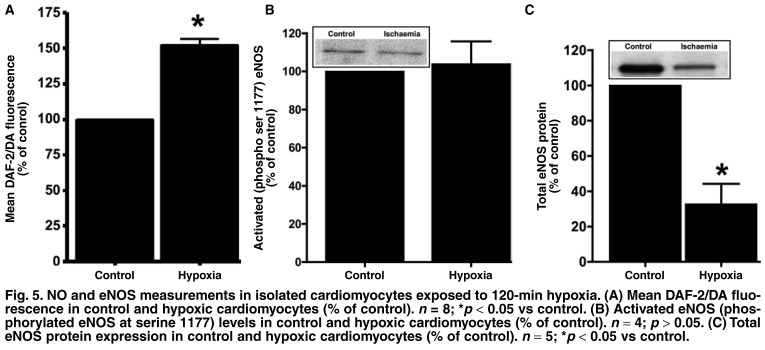
NO and eNOS measurements in isolated cardiomyocytes exposed to 120-min hypoxia. (A) Mean DA F-2/DA fluorescence in control and hypoxic cardiomyocytes (% of control). *n* = 8; *p < 0.05 vs control. (B) Activated eNOS (phosphorylated eNOS at serine 1177) levels in control and hypoxic cardiomyocytes (% of control). *n* = 4; *p* > 0.05. (C) Total eNOS protein expression in control and hypoxic cardiomyocytes (% of control). *n* = 5; *p < 0.05 vs control.

It has been suggested that the increased NO production is dependent on the duration or severity of the ischaemic insult, and that levels drop again as the duration of ischaemia increases.^3^ These observations may be attributed to NOS dysfunction/degradation as a result of increased ischaemia-induced cellular acidosis.^30^ However, in our cardiomyocyte models, we observed sustained high levels of NO at one^29^ and two hours of hypoxia (despite loss of eNOS expression in the latter; see Fig. 5A, C) using a relatively potent hypoxia protocol (ischaemic pelleting: cells centrifuged into a pellet and a remaining thin layer of supernatant sealed off with mineral oil). This observation may be due to eNOS-independent generation of NO (such as iNOS or nitrite reduction).

## NOS isoform involvement in hypoxia and ischaemia: iNOS and nNOS

There are enough data available to suggest that the increased NO production observed during hypoxia and ischaemia is at least in part due to increased NOS activity,^3^ and since all three major isoforms are expressed in cardiac tissue, iNOS, nNOS or eNOS could alone, or in combination, contribute to increased NO levels. However, there are few studies that characterise and/or directly measure the NOS isoforms involved in cardiac hypoxia and ischaemia.^3^

A number of studies have suggested the involvement of iNOS. The ‘NO hypothesis of delayed ischaemic preconditioning (IP)’^31^ proposes a crucial (cardioprotective) role for ischaemia-induced iNOS expression.^32^ Delayed IP is a phenomenon whereby an initial stimulus consisting of brief episodes of ischaemiareperfusion can lead to protection against a subsequent period of sustained ischaemia (induced three to four days later). According to the proposed mechanism, the initial ischaemia-reperfusion protocol triggers the release and activation of several factors and signalling pathways that eventually induce iNOS expression during the sustained ischaemic period, leading to NO generation. Others have also shown involvement of iNOS in hypoxia and ischaemia (in the non-preconditioned setting) by observing increased iNOS mRNA and protein expression.^33^-^35^ In rat cardiomyocytes, hypoxia induced iNOS expression via an HIF-1 (hypoxia inducible factor-1)-mediated iNOS gene-activating mechanism.^36^

Data from our laboratory also suggested a possible role for iNOS as one of the sources of increased NO levels in hypoxic cardiomyocytes, but not in cardiac microvascular endothelial cells.^28^,^29^ In another study on eNOS-/- knock-out mouse hearts, iNOS induction was greatly increased during ischaemia-reperfusion (probably as a compensatory mechanism) and the subsequent elevated NO levels were associated with cardioprotection. 37 The role of nNOS in hypoxia and ischaemia has also been generating some interest in recent years since it was discovered that nNOS was also expressed in cardiomyocytes and not only in the nerve terminals that supply the heart.^38^ Some studies have shown increased nNOS mRNA and protein expression during ischaemia,^34^,^39^ whereas others observed decreased expression.^40^

It is clear from the literature that more research needs to be done to elucidate the distinct role of nNOS, in particular in cardiac hypoxia and ischaemia. The role of the third isoform, eNOS, is discussed separately in the next section.

## NOS isoform involvement in hypoxia and ischaemia: eNOS

Despite the fact that eNOS is the most abundant and widely distributed NOS isoform in the heart^16^ and the predominant source of NO in the physiological setting,^2^ the regulation and activation of eNOS in the heart, particularly in hypoxia and ischaemia, remain relatively under-investigated.^15^,^36^ Most studies that did investigate these aspects of eNOS in conditions of low oxygen supply generally reported either retention or increased expression of eNOS protein, or increased activation. Normoxic control of eNOS protein expression was maintained in ischaemic cardiomyocytes^41^ and isolated hearts,^34^ whereas increased expression was observed in coronary endothelial cells^42^,^43^ and isolated hearts.^44^,^45^ A few studies also investigated the effects of ischaemia on activated eNOS levels. Elevations in ischaemia-induced levels of activated eNOS were observed in cardiac endothelial cells,^43^,^46^ cardiomyocytes^46^ and isolated hearts.^46^

On the whole therefore, it seems as if eNOS is a significant source of hypoxia/ischaemia-induced NO production in the heart, despite its traditional association with NO supply in the basal, physiological setting. We have recently provided further evidence that hypoxia/ischaemia is strongly associated with increased NO production and elevated levels of activated eNOS in isolated whole heart models (15 min global ischaemia; Fig. 4A, B) as well as in isolated cardiomyocytes and cardiac microvascular endothelial cells (CMECs).^29^ In these studies, measurement of eNOS phosphorylated at the serine 1177 residue was used as indicator of activated eNOS, since phosphorylation at this site has been shown to be a major activation mechanism of the enzyme.^11^,^47^

In order to establish a link between oxygen deprivation and eNOS activation, we investigated the role of the phosphatidylinositol-3 kinase (PI-3 K)–protein kinase B (PKB/Akt) pathway as a putative mediator.^29^ It is well known that activated PKB/Akt is the most prominent kinase responsible for ser1177 phosphorylation of eNOS^47^ and a previous study on porcine coronary endothelial cells demonstrated that hypoxia activated eNOS via PKB/Akt activation.^43^

**Fig. 6.**
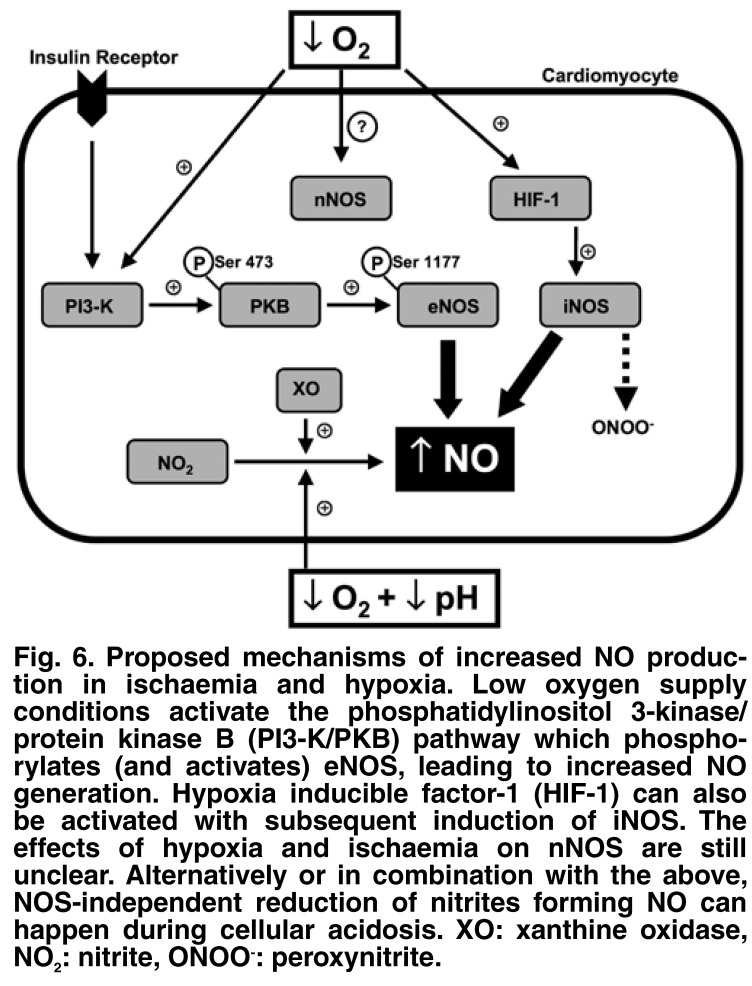
Proposed mechanisms of increased NO production in ischaemia and hypoxia. Low oxygen supply conditions activate the phosphatidylinositol 3-kinase/protein kinase B (PI3-K/PKB) pathway which phosphorylates (and activates) eNOS , leading to increased NO generation. Hypoxia inducible factor-1 (HIF-1) can also be activated with subsequent induction of iNOS . The effects of hypoxia and ischaemia on nNOS are still unclear. Alternatively or in combination with the above, NOS-independent reduction of nitrites forming NO can happen during cellular acidosis. XO: xanthine oxidase, NO_2_: nitrite, ONOO^-^: peroxynitrite.

Our data showed that PKB/Akt was activated by hypoxia (phosphorylation of serine 473 residue) and inhibition of PI-3 K–PKB/Akt activity resulted in decreased NO production during hypoxia in both cardiomyocytes and CMECs.^29^ Subsequently, it was shown in the cardiomyocytes that activated eNOS returned to control levels after PI-3 K–PKB/Akt pathway inhibition. In summary, the above data are the result of one of the first studies in which evidence is provided that activated cardiac eNOS (phospho eNOS ser1177) levels increase in cardiac hypoxia and ischaemia in the whole heart, cardiomyocytes and CMECs, and that activated PKB/Akt is an important mechanistic link through which hypoxia activates eNOS to generate NO (Fig. 6).

An interesting observation was made in a study on isolated perfused rat hearts in which it was shown that eNOS protein expression increased slightly (albeit not significantly) above control levels after 30 min of ischaemia, but then decreased significantly when ischaemia was extended to 60 and 90 min.^30^ It can be concluded from these results that prolonged ischaemia reduced eNOS expression in these hearts, possibly due to enzyme loss following degradation or due to loss of membrane integrity.

In our laboratory, both total eNOS protein and activated eNOS levels were measured in isolated perfused rat hearts subjected to global ischaemia (GI). The results showed that activated eNOS levels (phosphorylated eNOS at serine 1177) were significantly elevated at 15 min and then decreased to below control levels at 20 min GI (Fig. 4B), despite unchanged protein expression after 15 and 20 min of GI (Fig. 4C). Investigations on isolated cardiomyocytes in our laboratory showed similar trends: when exposed to 60 min of hypoxia, eNOS expression remained unchanged, and activated eNOS levels increased significantly.^29^ However, when hypoxia duration was increased to 120 min, we observed a significant loss of eNOS protein (Fig. 5C) associated with unchanged activated eNOS (Fig. 5B).

Therefore, from the above studies, it seems that cardiac eNOS is susceptible to the duration or severity of ischaemia, which is manifested by either reduced protein or decreased activated enzyme levels. Interestingly, our results showed that the elevated NO levels were sustained in both whole heart (Fig. 4A) and cardiomyocyte models (Fig. 5A), despite the increased duration of ischaemia/hypoxia and the subsequent negative effects on the ability of eNOS to produce NO. These observations underline the significance of the role of eNOS-independent sources of NO production such as iNOS and/or nNOS, and the increasing relevance of data flowing from studies investigating the reduction of nitrites to form NO (Fig. 6).^21^

With the advent of genetically modified animal models (i.e. gene knock-out, gene over-expression or gene silencing), the distinct roles of the NOS isoforms can be more specifically investigated. Some studies have investigated the role of eNOS in ischaemia and hypoxia by implementing gene modification.^3^ In studies on eNOS^-/-^ knock-out mouse hearts, contractile dysfunction associated with ischaemia-reperfusion injury was more pronounced,^48^,^49^ whereas in eNOS over-expressed mouse hearts, an improvement in contractile function was observed,^50^ pointing to a protective role for eNOS.

Greater myocardial injury (measured by determination of infarct size) was observed in eNOS-/- knock-out mouse hearts after exposure to ischaemia,^51^-^53^ whereas other data pointed to a reduction in infarct size.^53^ Interestingly, in a study on isolated, perfused hearts of eNOS-/- mice, it was observed that ischaemia was associated with increased NO production in what appeared to be a compensatory super-induction of the iNOS isoform. Myocardial injury in these hearts was significantly attenuated compared to wild-type hearts,^37^ pointing to a cardioprotective role for iNOS-derived NO in this scenario.

## Hypoxia/ischaemia-induced NO production: mostly protective, but some evidence of harmful effects

As mentioned earlier, most authors seem to agree that the increased NO production observed in hypoxia and ischaemia is protective. The cardioprotective properties of NO produced during hypoxia/ischaemia are particularly well demonstrated in the context of ischaemic preconditioning (IP).^32^ In preconditioned hearts, it was shown that endogenously produced NO can elicit protection, particularly in the delayed form of IP protection. The protection observed with IP has a bimodal pattern: an early wave of protection observed within minutes or hours of the experimental intervention (early IP), and a second, delayed and clinically more relevant wave of protection three to four days later (delayed IP).^54^

Also of great clinical interest is the plethora of studies that clearly demonstrate a cardioprotective role for either endogenous or exogenous NO in hearts subjected to ischaemia-reperfusion injury alone, in the absence of any preceding preconditioning interventions in both *in vivo* and *in vitro* models.^22^ A comprehensive review published in 2001, in which 92 previous studies were analysed, showed that 73% of them found a protective role for NO against ischaemia-reperfusion injury.^22^

There are several proposed cellular mechanisms through which NO is believed to elicit protection. These include increased cGMP synthesis (the ‘classical’ NO signalling pathway),^13^ putative antioxidant properties,^55^ mitochondrial K_ATP_ channel activation,^56^ inhibition of mitochondrial permeability transition pore (MPTP) opening,^3^ inhibition of mitochondrial oxidation,^13^ etc. In this regard, the role of the cGMP/PKG pathway has been increasingly acknowledged as a mechanism of the infarct-limiting effects of IP, ischaemic postconditioning, administration of exogenous NO donors, statins, etc in ischaemia-reperfusion injury models.^57^ PKG, which has been described as one of the ‘survival kinases’, could elicit protective effects by regulating Ca^2+^ homeostasis (modification of SR Ca^2+^ uptake) and activating K_ATP_ channel opening.^57^ The resulting protective biological effects observed are:

● cardiac circulation: increased vasodilation (and therefore improved coronary perfusion), decreased platelet aggregation, increased anti-inflammatory state, decreased endothelial dysfunction
● cardiac cells: decreased apoptosis, decreased oxygen consumption
● myocardium: increased ischaemic tolerance, improved contractile function and relaxation, reduced infarct size, decreased stunning, decreased arrhythmias.

However, there are also studies, using a variety of different models and protocols that could either not demonstrate protection58 or observed harmful effects.^35^,^59^,^60^ Results from investigations in our laboratory in a model of isolated cardiomyocytes pointed to a harmful role for NO released during a two-hour hypoxia protocol.^61^ It is generally accepted that harmful effects are often not due to NO per se, but rather a result of more reactive metabolic byproducts (ONOO-, NO_2_^•^ and OH^•^) formed when high amounts of NO are generated (e.g. due to pathophysiological induction of iNOS).^13^

The generation of ONOO- over longer periods of time can give rise to nitrosative stress, which is a term used to collectively describe the cytotoxic actions of ONOO^-^ via oxidation (and often destruction) of cellular components leading to, inter alia, dysfunctional signalling pathways, apoptosis and necrosis.^62^ The ability of NO or ONOO^-^ to directly combine with target proteins (nitrosylation, nitration) and thereby altering protein function (e.g. formation of nitrotyrosine), is therefore a mechanism that could result in deleterious effects.^63^,^64^ Harmful biological effects resulting (directly or indirectly) from NO in the pathophysiological setting include: poly-ADP ribose synthase (PARP) activation, matrix metalloproteinase (MMP) activation, DNA strand-breaks, thiol oxidation, increased apoptosis, increased necrosis, and decreased β-adrenergic responsiveness (manifesting as contractile dysfunction).^3^,^13^

As explained earlier, the beneficial versus harmful role of NO depends to a large extent on the specific NOS isoform involvement. A general rule of thumb is that eNOS-derived NO exerts protective effects during ischaemia and hypoxia. There seems to be convincing evidence that iNOS is associated with protective effects, particularly in the context of delayed IP; however, iNOS is also equally known to be involved in deleterious actions via the formation of ONOO^-^.^13^ With regard to the role of nNOS, too few studies are available as yet to draw any conclusions.

## Conclusion

The role of NO as a major signalling molecule in the heart and blood vessels is important and very relevant, not only to the field of basic medical sciences but also to clinical cardiology. Most experts agree that NO is by and large a potent and effective agent of cardioprotection. Therefore, the metabolism and cellular mechanisms underlying the various biological actions of NO should continue to receive the research attention they deserve, as this molecule has great potential as a future therapeutic modality in the prevention and treatment of ischaemic heart disease.
